# Households' Food Insecurity and Their Association With Dietary Intakes, Nutrition-Related Knowledge, Attitudes and Practices Among Under-five Children in Gaza Strip, Palestine

**DOI:** 10.3389/fpubh.2022.808700

**Published:** 2022-02-25

**Authors:** Abdel Hamid El Bilbeisi, Ayoub Al-Jawaldeh, Ali Albelbeisi, Samer Abuzerr, Ibrahim Elmadfa, Lara Nasreddine

**Affiliations:** ^1^Department of Nutrition, School of Medicine and Health Sciences, University of Palestine, Gaza Strip, Palestine; ^2^Regional Office for the Eastern Mediterranean (EMRO), World Health Organization (WHO), Cairo, Egypt; ^3^Health Research Unit, Palestinian Ministry of Health, Gaza Strip, Palestine; ^4^Visiting Scholar With the School of Public Health, Department of Social and Preventive Medicine, University of Montreal, Montréal, QC, Canada; ^5^Department of Nutritional Sciences, Faculty of Life Sciences, University of Vienna, Vienna, Austria; ^6^Nutrition and Food Sciences Department, Faculty of Agriculture and Food Sciences, American University of Beirut, Beirut, Lebanon

**Keywords:** anthropometric measurements, dietary intakes, food insecurity, nutrition-related knowledge, under-five children

## Abstract

The present study aimed to determine the households' food insecurity and their association with dietary intakes, nutrition-related knowledge, attitudes and practices (KAP) among under-five children in the Gaza Strip, Palestine. This cross-sectional study was conducted in 2021 among a representative sample of under-five children. A total of 350 children and their proxy were selected from all Gaza strip governorates, using a cluster random sampling method. The Radimer/Cornell food-security scale was used. A 24-h dietary recall was employed for dietary intakes assessment. Nutrition-related KAP for feeding under-five children, anthropometric measurements, and demographic-socioeconomic characteristics were obtained with an interview-based questionnaire. Statistical analysis was performed using SPSS version 25. The children from food-insecure households had a high prevalence of moderate underweight (30.4%), stunting (32.8%), wasting (9.6%), and acute undernutrition (30.4%). Between food-insecure and food-secure groups there were significant differences in weight, height/length, mid-upper arm circumference, weight-for-age and mid-upper arm circumference z-scores, underweight, acute undernutrition, intakes of protein, fat, vitamin D, zinc, continued breastfeeding, having nutrition-related adequate knowledge, having nutrition-related positive attitudes, and achieved minimum dietary diversity score (*P* < 0.05 for all). Additionally, about (56.0%) food-insecure households have inadequate nutrition-related knowledge, (77.6%) have nutrition-related negative attitudes, and (95.2%) did not achieve a minimum dietary diversity score. In conclusion, the children from food-insecure households had a high prevalence of moderate underweight, stunting, wasting, and acute undernutrition. Moreover, low economic status, poor dietary intakes, low levels of nutrition-related KAP, and lack of dietary diversity could contribute to the high levels of food insecurity among under-five children.

## Introduction

Food insecurity is a significant nutritional issue worldwide and is commonly found in low- and middle-income countries (1, 2). Since 2014, the global incidence of moderate or severe food insecurity has steadily increased, with the projected increase in 2020 equaling the preceding 5 years combined (3). In addition, in 2020, it is estimated that 22.0 % (149.2 million) of children under 5 years of age were affected by stunting, 6.7 % (45.4 million) were suffering from wasting, and 5.7 % (38.9 million) were overweight (4). Furthermore, it was projected that 119 million children under five would be stunted in 2030 in the 135 low- and middle-income countries (5). In the Gaza Strip, Palestine, over 68 % of households (about 1.3 million people) are severely or moderately food-insecure, according to the preliminary findings of the latest Socio-Economic and Food Security Survey carried out in 2018 (6). Also, stunting (10.3%) remains the most prevalent form of undernutrition among children under 5 years, followed by underweight (2.5%) and wasting (2.4%) (7). Moreover, a nutrition needs assessment was conducted in the most vulnerable areas in the Gaza strip, indicating that only 14% of children under 5 years of age had a minimum acceptable diet (8).

Food insecurity exists when people do not have adequate physical, social and economic access to sufficient, safe and nutritious food that meets their dietary needs and preferences for an active and healthy life (9). It is a global problem, and it is related to macro-and micronutrient deficiencies and lack of dietary diversity (10). Food insecurity has the potential to be harmful to individuals of any age, but it can be especially devastating to under-five children (11). Children who experience food insecurity may suffer from poor health and nutritional deficiencies (12). Inadequate nutrition can permanently alter children's brain architecture and stunt their intellectual capacity, affecting children's learning, social interaction and productivity (13). Children struggling with food insecurity may be at greater risk for stunted development, anemia, asthma, oral health problems, and hospitalization (14). Overall, food insecurity is linked with a poorer physical quality of life, preventing children from fully engaging in daily activities (15, 16). Effective solutions to child food insecurity require addressing the immediate food needs of individual households as well as the underlying economic factors contributing to local food insecurity (17, 18).

On the other hand, having physical and economic access to food on their own is insufficient to ensure that people are food-secure and well nourished. It is essential that people understand what constitutes a healthy diet, mainly what nutrition-related health issues affect their communities and how to address these through food-based approaches, and know how to best use their resources. They should also have positive attitudes toward nutrition, foods and closely related health issues to perform optimal dietary and feeding practices that ensure their nutritional wellbeing and that of their families (19, 20). In the present study, the Food and Agriculture Organization of the United Nations (FAO) questionnaire for assessing nutrition-related knowledge, attitudes and practices (KAP) feeding children younger than 5 years was used (21).

Additionally, nutrition-related knowledge and attitudes are necessary for dietary changes toward a healthier dietary pattern. For that reason, nutrition-related KAP is one of the key factors to achieving households' food and nutritional security. To the best of our knowledge, no study has ever investigated this association among under-five children in the Gaza Strip, Palestine. Therefore, the present study aimed to determine the households' food insecurity status and their association with dietary intakes, nutrition-related KAP among under-five children.

## Methods

### Study Design

This cross-sectional community-based study was conducted in 2021 among a representative sample of under-five children in the Gaza strip governorates. A total of 350 children and their proxy were selected from all Gaza strip governorates, using a cluster random sampling method. Households having at least one child (male or female), aged < 5 years, and living with his/her mother in the same household, and mothers and fathers aged ≥ 18 years and having under-five children were included in the present study. On the contrary, households without under-five children, under-five children with disabilities or chronic disease, preterm infants (<37 weeks), infants of diabetic mothers, and under-five children who have a history of complications during delivery (aspiration or trauma) were excluded from the present study.

### Study Location

The current study was conducted in the households of the Gaza strip, Palestine. The estimated population of the Gaza strip is about 2,106,745 million (22). The Gaza strip is divided into five governorates: North-Gaza, Gaza, Middle-Area, Khanyounis, and Rafah; with a population density of 19.3, 34.9, 14.4, 19.1, and 12.2%, respectively (23).

### Sample Size and Sampling Technique

In the present study, the representative sample size was calculated using the following formula (24).


(1) $$\begin{array}{l} Sample\;size\;(n)\;=\;\frac{Z_{1\;-\;\alpha /2}^{2}\;P(1\;-\;P)}{d^{2}}\\ \;=\frac{{\left(1.96\right)}^{2}(0.30)\;(1\;-\;0.30)}{{\left(0.05\right)}^{2}}\;=\;323 \end{array}$$

Where, Z_1−α/2_ = Standard normal variate (Z value is 1.96 for a 95 percent confidence level); *p* = Response distribution (30%); and *d* = Margin of error (5%).

Accordingly, the calculated sample size of the current study was 323 under-five children, to which we added 10% as an expected non-response rate. Finally, this study applied a cluster random sampling method to 350 under-five children and their proxy were responded and recruited. The sample was distributed into the five governorates of the Gaza strip based on the population density as follows: 42 from Rafah, 68 from Khanyounis, 50 from Middle-Area, 122 from Gaza, and 68 from North Gaza.

## Data Collection

### Interview Questionnaire

An interview-based questionnaire was used; the data was collected from the head of the household (mothers or fathers) and the under-five children by ten qualified data collectors, who were trained and prepared by the researcher. The questionnaire contains items about demographic and socio-economic characteristics of under-five children, the Radimer/Cornell food security scale (25), two non-consecutive days of the 24-h dietary recalls, anthropometric measurements, and the FAO nutrition-related KAP for feeding children younger than 5 years (26). Before data collection, a pilot study was carried out in thirty participants to enable the researcher to examine the tools of the study. The questionnaire and data collection process were modified according to the results of the pilot study.

### Demographic and Socio-Economic Characteristics of Under-five Children

An individual face-to-face interview was conducted with the heads of households (mothers or fathers) to collect information about demographic and socio-economic characteristics of under-five children, including gender, date of birth, gestational age (weeks), birth weight (kg), governorate, the nature of the living area, history of any disease, history of gestational diabetes, use of medications; household monthly income (NIS), and educational level of the heads of households (mothers or fathers). The age of the children (months) was calculated from their date of birth (from birth certificates) to the day of data collection. Additionally, the used categories of educational level and household monthly income (NIS) variables in the current study were similar to which mentioned in earlier studies in Gaza strip (27, 28).

### Food Insecurity Measurement

The 10-items Radimer/Cornell food security scale was used for determining the households food security status (25). The scale is a valid and reliable tool for measuring household food insecurity in a culturally diverse setting (29, 30). The households were classified by food security status as follow: (1) Household food secure: Negative answers to all hunger and food insecurity items; (2) Household food insecurity: Positive answers (“sometimes true” or “often true”) to one or more hunger and food insecurity items (29, 30).

### Dietary Intakes Assessment of Under-five Children

Two non-consecutive days of 24-h dietary recalls were employed to determine the quantity of macronutrients and micronutrients consumed by the under-five children. Mothers were requested to recall all beverages, number of breastfeeds, and food consumed by their children in the past 24 h. The portion sizes were estimated using a set of household measurements (i.e., plates, cups, glasses, and spoons). Dietary data from the 24-h dietary recall was processed by hand (office work) in order to calculate the net grams of foods consumed by the under-five children. This information was analyzed using the Nutritionist Pro Software version 7.1.0 (Axxya Systems, USA) (31) to determine energy (kcal) and nutrients intakes, including protein (g), carbohydrate (g), fat (g), iron (mg), vitamin A (μg), vitamin D (μg), calcium (mg), and zinc (mg).

## Anthropometric Measurements of Under-Five Children

### Length/Height and Weight

Recumbent length (cm) was recorded to the nearest 0.1 cm using the length board, appropriate for children under 2 years old. The mothers were asked to lay their children on their backs against the fixed headboard, compressing the hair and eyes looking straight up. In addition, the height (cm) and weight (kg) of children were measured following standard recommended procedures (32). A digital weighing scale (to the nearest 0.1 kg) (SECA, Germany) and a body meter (with the precision of 0.1 cm) (SECA, Germany) were used. The measurement for each child was carried out twice, and the average reading was documented as the final reading. Furthermore, the age, weight, and height of the children were translated into three indices: Height/length for age (HAZ), weight for age (WAZ), and weight for height/length (WHZ), which were expressed in terms of z-score using the WHO Anthro Software (Version 3, 2009) (33). Then, the under-five children were classified into moderate and severely underweight, moderate and severe stunting, and moderate and severe wasting, which mean that WAZ, HAZ, and WAZ z-scores are < −2 and < −3, respectively (32).

### Mid-upper Arm Circumference

MUAC (cm) was recorded to the nearest 0.1 cm using the MUAC measuring tape. Investigators measured the MUAC at the midpoint of the arm where the measuring tape was snugged to the skin but not pressing soft tissues (34). The measurement for each child was carried out twice, and the average reading was recorded as the final reading. Then, the measurement of MUAC was used to calculate MUAC for age z-scores (MUACZ), using the WHO Anthro Software (version 3, 2009) (33). Moreover, the children were classified into moderate and severe acute undernutrition as MUACZ is < −2 and < −3, respectively (35).

### Assessment of Nutrition-Related KAP

The FAO of the United Nations questionnaire for assessing nutrition-related KAP was used to conduct high-quality nutrition-related KAP surveys. The questionnaire comprises predefined questions that capture information on critical KAP related to feeding children younger than 5 years (26). In the present study, the nutrition-related KAP consists of 7-questions related to nutrition-related knowledge, 7-questions related to nutrition-related attitudes, and other questions about nutrition-related practices and dietary diversity.

### Calculation of the Minimum Dietary Diversity Score

The minimum nutritional diversity indicator was calculated as the number of under-five children who received food from four or more food groups during the previous day, divided by the number of under-five children, multiplied by 100; breast milk was not included among the food groups (26).

### Data Analysis

The Statistical Package for Social Science (SPSS) for Windows (version 25) was used for data analysis. Descriptive statistics were used to describe continuous and categorical variables. The chi-square test and fisher's exact test were used to determine the significant differences between categorical variables. The differences between means were tested by independent samples *t*-test. A *P* < 0.05 was considered as statistically significant.

## Results

A total of 350 under-five children and their proxy were included in the present study (54.8% were males, and 45.2% were females). The characteristics of under-five children were compared by household food insecurity status (Table 1). The results revealed that children in food-insecure households had a higher proportion (71.4%) than those in food-secure households (28.6%). The mean ages (months) for children in food-secure and food-insecure households were 34.05 ± 12.16 and 32.26 ± 13.21, respectively. There were significant differences in the governorate of residence and the nature of residence area between children in food-secure and food-insecure households (*P*-values = 0.001, and 0.046, respectively), as half of the study participants (50.2%) residing in refugee camps. In addition, a significant difference was found in monthly income (NIS) between food-secure and food-insecure households (*P*-value = 0.009).

**Table 1:** Demographic socioeconomic characteristics of under-five children by household food-security status (*n* = 350).

**Variables**	**Household food-secure (*n* = 100)**	**Household food-insecure** **(*n* = 250)**	***P*-value**
**Age (months)**			
Mean ± SD	34.05 ± 12.16	32.26 ± 13.21	0.431^a^
**Gender**			
Male**s**	56.0 (56.0)	134.0 (53.6)	0.792^b^
Females	44.0 (44.0)	116.0 (46.4)	
**Governorate**			
North Gaza	50.0 (50.0)	18.0 (7.2)	<0.001^b^^*^
Gaza	36.0 (36.0)	86.0 (34.4)	
Middle Area	8.0 (8.0)	42.0 (16.8)	
Khanyounis	4.0 (4.0)	64.0 (25.6)	
Rafah	2.0 (2.0)	40.0 (16.0)	
**Living area**			
City	36.0 (36.0)	90.0 (36.0)	0.046^b^^*^
Village	14.0 (14.0)	34.0 (13.6)	
Camp	50.0 (50.0)	126.0 (50.4)	
**Household monthly income (NIS)**			
≤ 2,000	76.0 (76.0)	228 (91.2)	0.009^b^^*^
> 2,000	24.0 (24.0)	22.0 (8.8)	
**Educational level of the head of households (mothers or fathers)**			
Low education	28.0 (28.0)	82.0 (32.8)	0.334^b^
High education	72.0 (72.0)	168 (67.2)	

a *Independent Samples t-test*.

b *Chi-Square Test*.

* *Difference is significant at the 0.05 level (two-tailed)*.

*NIS, New Israeli Shekel*.

*Low education: Illiterate, primary, or preparatory; High education: Secondary, or university*.

Table 2 shows the nutritional status and anthropometric measurements of under-five children by household food security status. The study showed that under-five children from food-insecure households had a lower mean z-score for all indexes, and a higher prevalence of moderate underweight, moderate stunting, moderate wasting, and moderate acute undernutrition, than their food-secure counterparts. Furthermore, the results demonstrated that, of food-insecure under-five children, about 76.0 (30.4%) were moderately underweighted, 82.0 (32.8%) moderately stunted, 24.0 (9.6%) moderately wasted, and 76.0 (30.4%) moderately acute undernourished. Moreover, there were significant differences in weight (kg), height/length (cm), mid-upper arm circumference (MUAC) (cm), weight-for-age z-score (WAZ), mid-upper arm circumference z-score (MUACZ), underweight, and acute undernutrition between the food-insecure and food-secure groups (*P* < 0.05 for all).

**Table 2:** Nutritional status and anthropometric measurements of under-five children by household food-security status (*n* = 350).

**Measurements**	**Household food-secure (*n* = 100)**	**Household food-insecure** **(*n* = 250)**	***P*-value**
**Weight (kg)**			
Mean ± SD	14.83 ± 3.59	12.95 ± 4.02	0.004^a^^*^
**Height/Length (cm)**			
Mean ± SD	74.72 ± 31.07	58.25 ± 40.81	0.012 ^a^^*^
**MUAC (cm)**			
Mean ± SD	17.56 ± 0.93	13.62 ± 2.86	<0.001^a^^*^
**WAZ (z-score)**			
Mean ± SD	0.51 ± 1.14	−0.51 ± 1.54	<0.001^a^^*^
**HAZ (z-score)**			
Mean ± SD	−1.28 ± 1.52	−1.34 ± 1.75	0.834^a^
**WHZ (z-score)**			
Mean ± SD	0.82 ± 1.26	0.38 ± 1.63	0.086^a^
**MUACZ (z-score)**			
Mean ± SD	1.46 ± 0.44	−1.22 ± 1.61	<0.001^a^^*^
**Underweight**			
Normal	100 (100)	174.0 (69.6)	<0.001^b^^*^
Moderate	0.0 (0.0)	76.0 (30.4)	
Severe	0.0 (0.0)	0.0 (0.0)	
**Stunting**			
Normal	82.0 (82.0)	168.0 (67.2)	0.064^**b**^
Moderate	18.0 (18.0)	82.0 (32.8)	
Severe	0.0 (0.0)	0.0 (0.0)	
**Wasting**			
Normal	96.0 (96.0)	226 (90.4)	0.355^c^
Moderate	4.0 (4.0)	24.0 (9.6)	
Severe	0.0 (0.0)	0.0 (0.0)	
**Acute undernutrition**			
Normal	100.0 (100)	174.0 (69.6)	<0.001^b^^*^
Moderate	0.0 (0.0)	76.0 (30.4)	
Severe	0.0 (0.0)	0.0 (0.0)	

*MUAC, Mid upper arm circumference; WAZ, weight for age z-score; HAZ, Height/length for age z-score; WHZ, weight for height/length z-score; MUACZ, Mid upper arm circumference for age z-score*.

*Moderate and severely underweight, moderate and severe stunting, and moderate and severe wasting, which mean that weight for age, height/length for age, and weight for height/length z-scores are < −2 and < −3, respectively (32). Moderate, and severe acute undernutrition as MUACZ is < −2 and < −3, respectively (35)*.

a *Independent Samples t-test*.

b *Chi-square test*.

c *Fisher's Exact Test*.

* *Difference is significant at the 0.05 level (two-tailed)*.

Table 3 shows the energy, macronutrients and micronutrients intakes among under-five children by household food security status. For children from food-insecure households the calculated mean intake for energy (kcal) amounted to 912 ± 217, protein (g) 42.36 ± 15.57, carbohydrate (g) 105.85 ± 29.79, fat (g) 30.97 ± 10.45, iron (mg) 4.48 ± 2.48, vitamin A RAE (μg) 307.96 ± 158.99, vitamin D (μg) 4.60 ± 3.39, calcium (mg) (456.13 ± 224.65), and zinc (mg) 3.05 ± 1.21. These were generally lower than those calculated for their counterparts from the food-secure group. Moreover, the calculated difference for protein (2.66 g), fat (3.4 g), vitamin D (1.31 μg) and zinc (0.81 mg) was significant (*P*-values = 0.041, 0.032, 0.036, and 0.044, respectively).

**Table 3:** Energy, macro and micronutrients intake among under-five children by food security status (*n* = 350).

**Variables**	**Household food-secure** **(*n* = 100)**	**Household food-insecure** **(*n* = 250)**	***P*-value**
**Energy (kcal)**			
Mean ± SD	913 ± 177	912 ± 217	0.982
**Protein (gram)**			
Mean ± SD	45.02 ± 14.76	42.36 ± 15.57	0.041^*^
**Carbohydrate (gram)**			
Mean ± SD	114.94 ± 43.74	105.85 ± 29.79	0.178
**Fat (gram)**			
Mean ± SD	34.37 ± 12.27	30.97 ± 10.45	0.032^*^
**Iron (mg)**			
Mean ± SD	4.72 ± 2.59	4.48 ± 2.48	0.579
**Vitamin A RAE (microgram)**			
Mean ± SD	310.43 ± 231.01	307.96 ± 158.99	0.940
**Vitamin D (microgram)**			
Mean ± SD	5.91 ± 3.81	4.60 ± 3.39	0.036^*^
**Calcium (mg)**			
Mean ± SD	465.60 ± 227.54	456.13 ± 224.65	0.805
**Zinc (mg)**			
Mean ± SD	3.86 ± 1.19	3.05 ± 1.21	0.044^*^

*Statistical testing using Independent samples t-test*.

* *Difference is significant at the 0.05 level (two-tailed)*.

*Vitamin A RAE, Vitamin A Retinol Activity Equivalents*.

**Figure 1:**
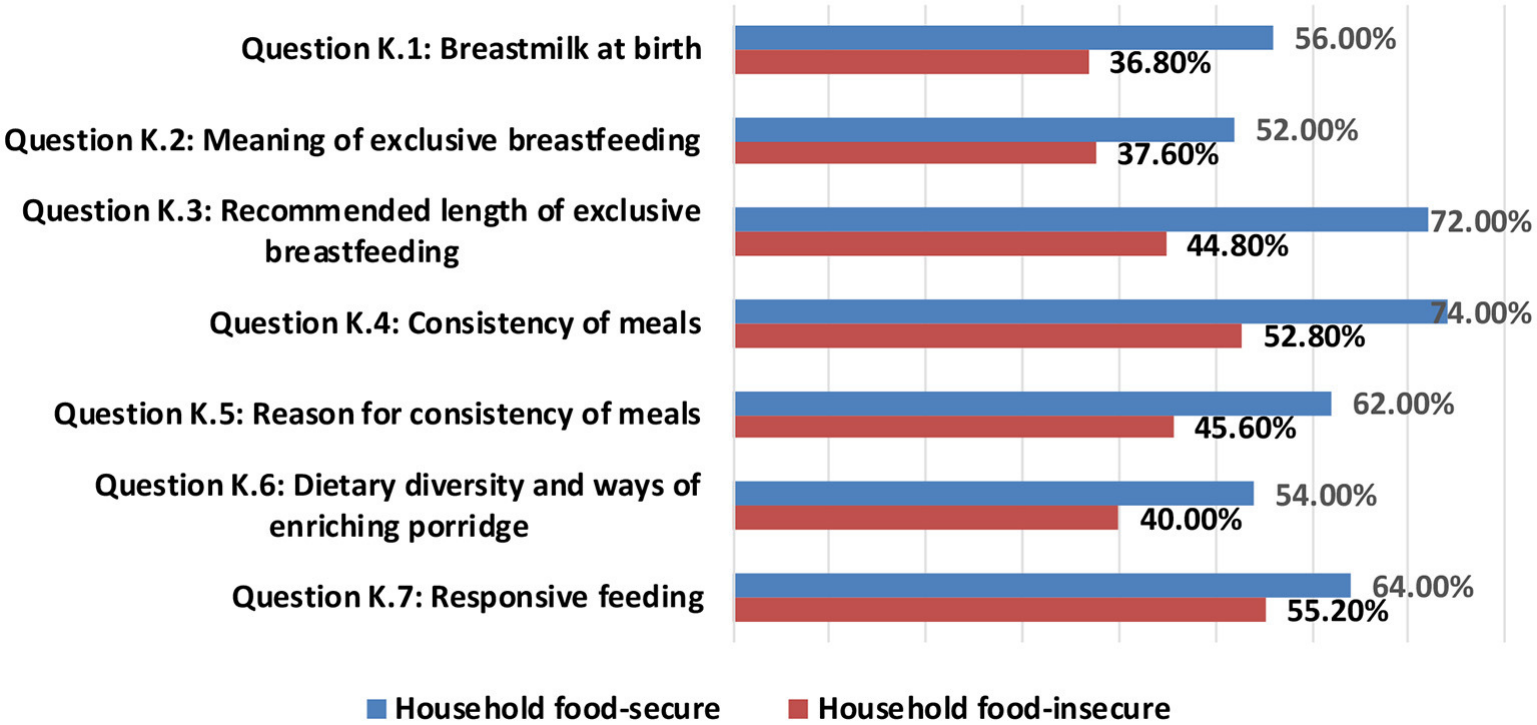
Nutrition-related adequate knowledge among household food-secure and household food-insecure.

Figure 1 shows the percentage of nutrition-related adequate knowledge for feeding under-five children among household food-secure and household food-insecure. The average percentages of seven dimensions of nutrition-related adequate knowledge were obtained from the head of the household (mothers or fathers) about their under-five children. The results revealed that the highest average percentage (74.0%) of nutrition-related adequate knowledge among food-secure households were in the dimension of “consistency of meals”; while the highest average percentage (55.2%) of nutrition-related adequate knowledge among food-insecure households were in the dimension of “responsive feeding”. The lowest average percentage of nutrition-related adequate knowledge among food-secure and food-insecure households was in the dimensions of “meaning of exclusive breastfeeding” and “breast milk at birth” of 52.0 and 36.8%, respectively.

**Figure 2:**
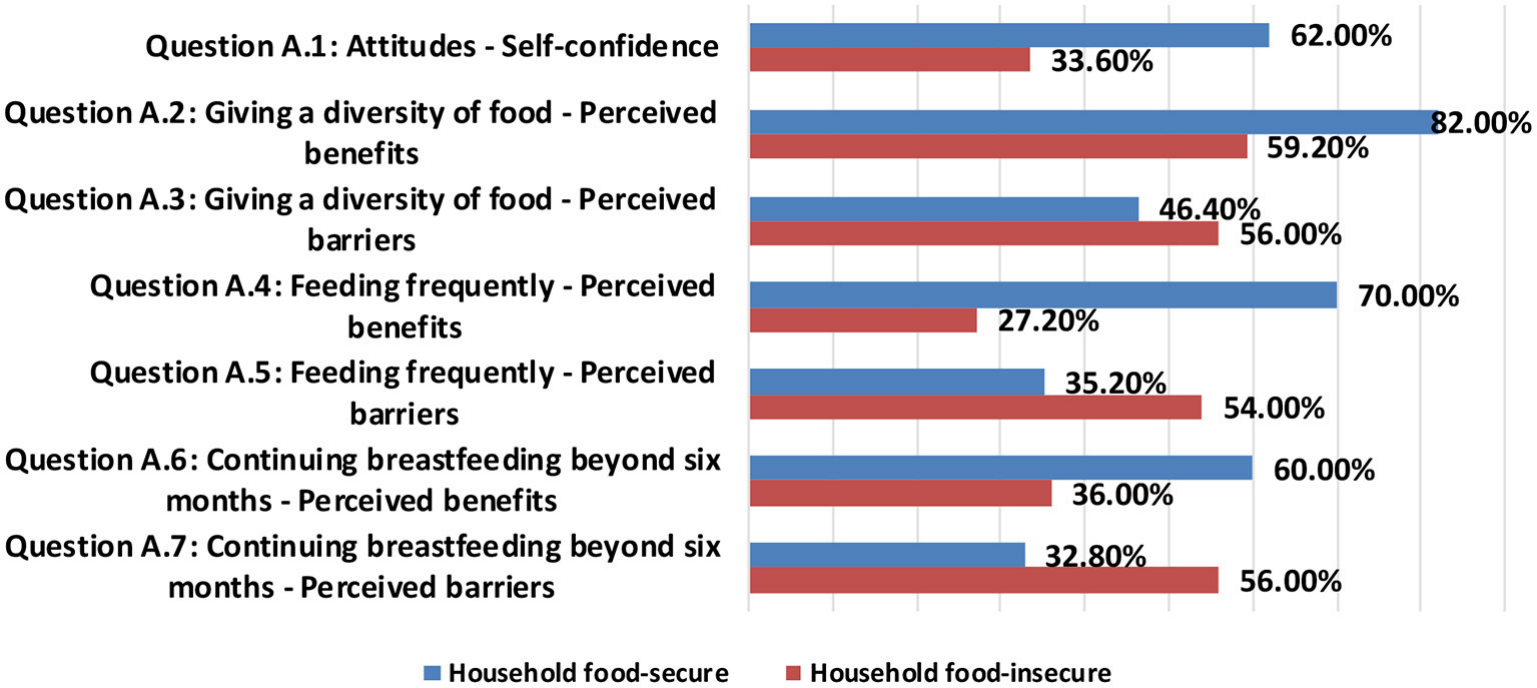
Nutrition-related positive attitudes among household food-secure and household food-insecure.

Figure 2 presents the average percentages of seven dimensions of nutrition-related positive attitudes for feeding under-five children among household food-secure and household food-insecure. The data were obtained from the head of the household (mothers or fathers) about their under-five children. The dimension of “Giving a diversity of food-perceived benefits” got the highest average percentage of nutrition-related positive attitudes among both food-secure and food-insecure households of 82.0 and 59.0%, respectively. Whereas, the lowest average percentage of nutrition-related positive attitudes for feeding under-five children among food-secure and food-insecure households were in the dimensions of “continuing breastfeeding beyond 6 months-perceived barriers” and “feeding frequently-perceived benefits” of 32.80 and 27.20%, respectively.

Table 4 shows the practices and the diet diversity of under-five children by household food security status. The study indicated the presence of significant differences among food-secure and food-insecure households regarding the dimensions of continued breastfeeding, intake of group 4: flesh foods, and group 7: other fruits and vegetables (*P*-value = 0.044, 0.013, and 0.011, respectively). About 72.8 and 54.0% of under-five children in food-insecure and food-secure households, respectively, do not continue breastfeeding (*P* < 0.05). In addition, 92.0 and 78.0% of under-five children in food-insecure and food-secure households do not consume group 4: flesh foods, respectively (*P* < 0.05). Furthermore, 38.4 and 20.0% of under-five children in food-insecure and food-secure households do not intake group 7: other fruits and vegetables, respectively.

**Table 4:** Practices and dietary diversity of under-five children by household food-security status (*n* = 350).

**Variables**	**Household food-secure (*n* = 100)**	**Household food-insecure** **(*n* = 250)**	***P*-value^**a**^**
**Continued breastfeeding**			
Yes	44.0 (44.0)	62.0 (24.8)	0.044^*^
No	54.0 (54.0)	182 (72.8)	
Don't know	2.0 (2.0)	6.0 (2.4)	
**Dietary diversity**			
**Group 1: Grains, roots, and tubers**			
Yes	44.0 (44.0)	106 (42.4)	0.842
No	56.0 (56.0)	144 (57.6)	
**Group 2: Legumes and nuts**			
Yes	44.0 (44.0)	90.0 (36.0)	0.327
No	56.0 (56.0)	160 (64.0)	
**Group 3: Dairy products**			
Yes	76.0 (76.0)	166 (66.4)	0.215
No	24.0 (24.0)	84.0 (33.6)	
**Group 4: Flesh foods**			
Yes	22.0 (22.0)	36.0 (8.0)	0.013^*^
No	78.0 (78.0)	230 (92.0)	
**Group 5: Eggs**			
Yes	68.0 (68.0)	71.0 (56.8)	0.115
No	32.0 (32.0)	54.0 (43.2)	
**Group 6: Vitamin A fruits and vegetables**			
Yes	42.0 (42.0)	74.0 (29.6)	0.082
No	58.0 (58.0)	176 (70.4)	
**Group 7: Other fruits and vegetables**			
Yes	80.0 (80.0)	154 (61.6)	0.011^*^
No	20.0 (20.0)	96.0 (38.4)	
**Others: Not counted in the dietary diversity score**			
Yes	22.0 (22.0)	62.0 (24.8)	0.695
No	78.0 (78.0)	188 (75.2)	
**Minimum meal frequency**			
Mean ± SD	2.85 ± 1.09	2.47 ± 1.25	0.053^b^

a *Statistical testing using Chi-Square Test*.

b *Independent Samples t-test*.

* *Difference is significant at the 0.05 level (two-tailed)*.

Table 5 shows the overall knowledge, attitude, and achieved minimum dietary diversity score of under-five children by household food security status. Significant differences were found among food-secure and food-insecure households regarding adequate nutrition knowledge and positive attitudes for feeding under-five children and achieved minimum dietary diversity scores (*P*-value = 0.001, 0.003, and 0.019, respectively). More than half of food-insecure households (56.0%) have inadequate nutrition-related knowledge for feeding under-five children, while only 28.0% of food-secure households have inadequate knowledge. Furthermore, about (77.6%) of food-insecure households have nutrition-related negative attitudes for feeding under-five children, while only (28.0%) of food-secure households have negative attitudes. Most food-insecure households (95.2%) did not achieve a minimum dietary diversity score, while 84.0% of food-secure households did not achieve a minimum dietary diversity score.

**Table 5:** The overall knowledge, attitudes, and achieved minimum dietary diversity score of under-five children by household food-security status (*n* = 350).

**Variables**	**Household food-secure (*n* = 100)**	**Household food-insecure** ** (*n* = 250)**	***P*-value^**a**^**
**Have nutrition-related adequate knowledge**			
Yes	72.0 (72.0)	110 (44.0)	0.001^*^
No	28.0 (28.0)	140 (56.0)	
**Have nutrition-related positive attitudes**			
Yes	72.0 (72.0)	56.0 (22.4)	0.003^*^
No	28.0 (28.0)	194.0 (77.6)	
**Achieved minimum dietary diversity score**			
Yes	16.0 (16.0)	12.0 (4.8)	0.019^*^
No	84.0 (84.0)	238 (95.2)	

a *Statistical testing using Chi-Square Test or Fisher's Exact Test*.

* *Difference is significant at the 0.05 level (two-tailed)*.

## Discussion

The current study is the first to investigate the issue of households' food insecurity and their association with dietary intakes, nutrition-related KAP among under-five children in the Gaza strip, indicating that household food insecurity was widespread among Gaza strip families. In this survey, approximately two-thirds of the under-five children were in food-insecure households. In line with the present findings, other surveys carried out in the Gaza strip stated a high prevalence of household food insecurity (6, 36). This indicates that food insecurity is a significant issue facing Palestinians in the Gaza strip. In addition, the present study showed that food insecurity is common in households with lower economic status. Consistent with this result, the UNICEF malnutrition conceptual framework reported that the poor financial situation of households negatively affects food access (37).

Our key results indicate that under-five children from food-insecure households had a higher prevalence of moderate underweight (30.4%), stunting (32.8%), wasting (9.6%), and acute undernutrition (30.4%). In addition, household food insecurity was associated with mean MUAC in children in terms of age z-scores, underweight, and acute undernutrition, but not with stunting and wasting. Studies conducted in the Gaza strip and developing countries indicated an association between household food insecurity and child underweight (38–42). Moreover, this result was in accordance with the UNICEF conceptual framework for malnutrition in developing countries; household food insecurity adversely affects the nutritional status of children by reducing the quantity and quality of food intake (37). Studies in developing countries have revealed no association between food insecurity, child wasting, and stunting (42–44), whereas other studies showed a positive relationship between food insecurity and stunting (43, 45, 46).

The current study showed that energy and macro-and micronutrients intakes, which contribute to crucial development indices of a growing child, among under-five children from food-insecure households were lower than among their food-secure counterparts. This result has been discussed and confirmed in the literature, as the poor economic status of households negatively affects the food expenditure and limits food choices to affordable options, which may not include required nutrient sufficiency for wellbeing (47–49). Moreover, this study has shown significant differences between food-secure and food-insecure households regarding having nutrition-related adequate knowledge and nutrition-related positive attitudes for feeding under-five children. More than half of food-insecure households (56.0%) have inadequate nutrition-related knowledge for feeding under-five children, while only 28.0% of food-secure households have inadequate knowledge. Furthermore, about (77.6%) of food-insecure households have nutrition-related negative attitudes, while only (28.0%) of food-secure households have negative attitudes. Nutrition-related knowledge and attitude are necessary for dietary changes toward a healthier dietary pattern. For that reason, food and nutrition-related KAP is one of the key factors to achieving household food and nutritional security (50). This study also revealed that achieved minimum dietary diversity score is significantly associated with household food security status. Most food-insecure households (95.2%) did not achieve a minimum dietary diversity score, while this this level was 84.0% for food-secure households. A Nepalese study supports this result – secondary data analysis of the Nepalese Demographic and Health Survey (51), and an Ethiopian study- Minimum dietary diversity and associated factors among children aged 6–23 months in Addis Ababa, Ethiopia (52). This can be attributed to the fact that children from food-secure households may eat various foods because their families may be more likely to afford miscellaneous foods than children from food-insecure households.

This study shares the standard limitation of cross-sectional design, challenging to make a causal association. Besides, information was collected from household heads (mothers/fathers) it was likely to have recall bias. Moreover, as the study considered only the 24-h recall method, it might not correctly reflect the exact figure of under-five children past dietary habits. Since anthropometric measurements are subject to measurement bias, we may not exclude some misclassified children's nutritional status classification. However, there was a standardization of measurement tools, intensive training of data collectors and careful supervision to overcome measurement errors and interview bias. Finally, although those diseased children were excluded, there may be some children who were not ill the week before but have lost their appetite, which may undervalue our results. However, this work should be seen as opening the door to the design of more rigorous studies on this phenomenon and alternatives that efficiently improve the capacity of under-five children caregivers.

## Conclusion

In conclusion, the children from food-insecure households had a higher prevalence of moderate underweight, stunting, wasting, and acute undernutrition. Moreover, low economic status, poor dietary intake, low levels of nutrition-related KAP, and lack of dietary diversity could contribute to the high levels of food insecurity among under-five children. Policy makers should continue to focus attention and investments in the most appropriate combinations of interventions to mitigate food insecurity levels among under-five children in the Gaza strip, Palestine.

## Data Availability Statement

The raw data supporting the conclusions of this article will be made available by the authors, without undue reservation.

## Ethics Statement

The study protocol was approved by the Palestinian Health Research Council (Helsinki Ethical Committee of Research Number: PHRC/HC/961/21), University of Palestine Ethical Committee of Research, the Palestinian Ministry of Health, and Ministry of Interior. Furthermore, informed consent was obtained from each participant or their proxy. Written informed consent to participate in this study was provided by the participants' legal guardian/next of kin.

## Informed Consent Statement

Written informed consent was obtained from each participant or their proxy.

## Author Contributions

AE collected, analyzed, and interpreted the data and wrote the first draft of the manuscript. AE, AA-J, AA, SA, IE, and LN significantly contributed in the study design and the critical review of the manuscript. AE and AA-J remarkably contributed to the analysis and interpretation of data and the critical review of the manuscript. All authors approved the final manuscript.

## Conflict of Interest

The authors declare that the research was conducted in the absence of any commercial or financial relationships that could be construed as a potential conflict of interest.

## Publisher's Note

All claims expressed in this article are solely those of the authors and do not necessarily represent those of their affiliated organizations, or those of the publisher, the editors and the reviewers. Any product that may be evaluated in this article, or claim that may be made by its manufacturer, is not guaranteed or endorsed by the publisher.
